# The Beneficial Effects of Morusin, an Isoprene Flavonoid Isolated from the Root Bark of *Morus*

**DOI:** 10.3390/ijms21186541

**Published:** 2020-09-07

**Authors:** Dong Wook Choi, Sang Woo Cho, Seok-Geun Lee, Cheol Yong Choi

**Affiliations:** 1 Department of Biological Sciences, Sungkyunkwan University, Suwon 16419, Korea; dongwookchoi85@gmail.com (D.W.C.); swcho0628@naver.com (S.W.C.); 2 Department of Science in Korean Medicine, Kyung Hee University, Seoul 02447, Korea; 3 KHU-KIST Department of Converging Science & Technology, Kyung Hee University, Seoul 02447, Korea

**Keywords:** morusin, antioxidants, natural products, cell signaling, inflammatory diseases, neurological disorders, diabetes, cancer

## Abstract

The root bark of *Morus* has long been appreciated as an antiphlogistic, diuretic and expectorant drug in Chinese herbal medicine, albeit with barely known targets and mechanisms of action. In the 1970s, the development of analytic chemistry allowed for the discovery of morusin as one of 7 different isoprene flavonoid derivatives in the root bark of *Morus*. However, the remarkable antioxidant capacity of morusin with the unexpected potential for health benefits over the other flavonoid derivatives has recently sparked scientific interest in the biochemical identification of target proteins and signaling pathways and further clinical relevance. In this review, we discuss recent advances in the understanding of the functional roles of morusin in multiple biological processes such as inflammation, apoptosis, metabolism and autophagy. We also highlight recent in vivo and in vitro evidence on the clinical potential of morusin treatment for multiple human pathologies including inflammatory diseases, neurological disorders, diabetes, cancer and the underlying mechanisms.

## 1. Introduction

The therapeutic relevance of the root bark of the mulberry tree (genus *Morus*) as an antiphlogistic, diuretic and expectorant drug has long been acknowledged in Chinese herbal medicine [1]. The rapid development of analytic chemistry in the 1970s led researchers to explore the constituents of this mysterious plant with remarkable clinical potential [2,3,4,5]. As a result of these efforts, a group at Toho university in Japan achieved the consecutive purification of two 2-arylbenzofuran derivatives and seven isoprene-substituted flavonoid derivatives from the benzene extracts of the root bark of a variety of plants in the genus *Morus* including *Morus alba* L. and *Morus nigra* L. Such discoveries, taken together with the anti-inflammatory properties of flavonoid species, significantly provoked scientific attention to the potential application of *Morus*-derived flavonoids to a myriad of human pathologies beyond its known benefits, leading to subsequent biochemical studies from multiple research groups in which the chemical and bioactive properties of the phytochemicals were tested.

**Figure 1:**
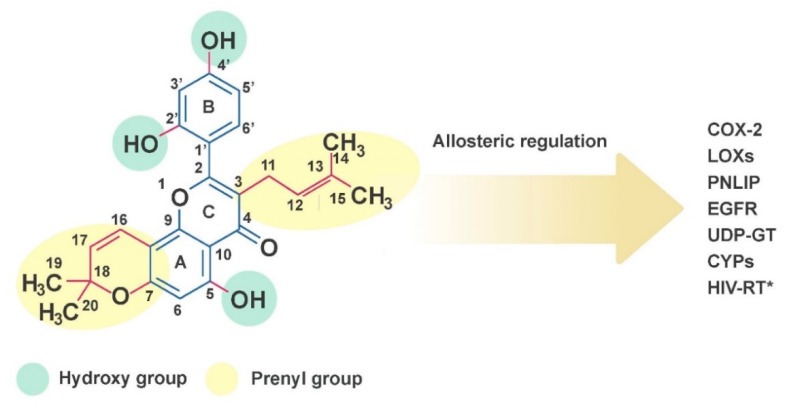
The chemical structure of morusin. Molecular targets of morusin include cyclooxygenase-2 (COX-2), lipoxygenases (LOXs), pancreatic lipase (PNLIP), epidermal growth factor receptor (EGFR), UDP-glucuronosyltransferase, cytochrome P450 (CYP) and HIV reverse transcriptase (* in silico analysis only).

Morusin is one of the *Morus*-derived flavonoids, which has been highlighted for its outstanding antioxidant capacity over the other flavonoids due to unique chemical and bioactive properties (Figure 1) [6]. Its versatile potential against human pathologies including cancer, immune dysfunction and metabolic disorders has been intensively tested in in vitro systems, although the underlying mechanisms and clinical evidence in vivo are yet to be fully explored. In this review, we describe the previously reported physiological processes induced by morusin, as well as in vitro and in vivo evidence connecting its therapeutic potential to relevant human pathologies including cancer. Finally, we highlight our recent discoveries of mechanisms regarding the strategies employed by cancer cells to resist morusin treatment and propose the cotreatment of morusin and relevant inhibitors as a potential tactic for boosting the anti-tumor capacity of the morusin.

## 2. Results

### 2.1. Chemical Properties of Morusin

Various classes of prenylated flavonoids have been isolated from the root bark of *Morus*. Among these, morusin has been highlighted for its versatile effects on human physiology and pathology. Morusin is a prenylated flavone with strong antioxidant capacity, which is structurally characterized by (1) a prenyl unit at position 3, (2) hydroxy groups at 5, 2′′ and 4′ and (3) a 2,2-dimethyl pyran group across positions 7 and 8 (Figure 1) [6]. The structure-bioactivity relationship was analyzed by the comparison of many prenylated flavonoids in the number and position of prenyl moieties [7,8,9,10]. In general, attachment of hydroxy group and prenyl group on the flavone backbone affects the bioactivities of a compound depending on the position and number of functional groups. Prenylation results in a more lipophilic compound to provide high affinity with the cell membrane, while leads to decreased bioavailability and plasma absorption [10]. The prenylations at the C-3 and C-7 position in the flavone backbone of morusin greatly contribute to its high cytotoxicity against murine P-388 cells [11,12]. However, the change from a cyclic form of the prenyl group at the C-8 position of morusin to a free prenyl group in kuwanon C slightly reduced the cytotoxic effects, while inhibitory activity against β-secretase and anti-bacterial activity against *E.coli* as well as *S. typhimurium* were markedly increased [8,9]. On the other hand, cyclization of the prenyl group at C-7 of morusin reduced inhibitory activity against tyrosinase and α-glucosidase [7].

Morusin may allosterically regulate several enzymes including cyclooxygenase-2, lipoxygenases, pancreatic lipase, epidermal growth factor receptor, UDP-glucuronosyltransferase, acetylcholine esterase (AChE), matrix metalloproteinases (MMP-9 and MMP-2), cytochrome P450 and HIV reverse transcriptase (further discussed in later sections) [13,14,15,16,17]. Such abilities have been decoupled from its antioxidant and anti-inflammatory capacities, since each carbon residue appears to play differential roles in mediating biological functions. For example, a prenyl substitution on C-3 of morusin caused noncompetitive inhibition characteristics toward AChE, while non-prenylated flavonoids showed mixed inhibition kinetics [18]. In addition, hydroxylation at C-5′ of morusin determined the selective inhibition of different oxygenases. Morusin, which lacks the 5′-hydroxyl group of artonin E, was a less potent 5-lipoxygenase inhibitor. Morusin displayed broad inhibitory activities against several lipoxygenases, while artonin E, produced by hydroxylation at C-5′ of morusin, displayed inhibitory activity against 5-lipoxygenase higher by one order of magnitude [19]. Molecular docking analysis between morusin and the cytochrome isoform CYP3A4 indicated that oxygens in the pyran ring, C-5 and C-2′ are involved in hydrogen bonds with CYP3A4 and the B ring structure of morusin is involved in π-π interaction with Phe108 of CYP3A4 [16,20]. Hydrogen bond formation between the 2′-hydroxy group of morusin and Tyr393 of MMP-9 and cation-pi interactions between the flavone backbone of morusin and Tyr423 of MMP-9 were also associated with morusin binding to MMP-9 [21]. Docking analysis of morusin with 5-lipoxygenase (5-LOX) indicated that the 2′- and 4′-hydroxy groups of the B ring structure of morusin form hydrogen bonds with Val127 of both the B and F chain in 5-LOX, respectively. Moreover, the 3-prenyl group of morusin aligned into the hydrophobic groove generated by Val127, Ala128, Leu124, Leu135 and Phe131 of the 5-LOX active site [22], indicating that hydroxylation and prenylation of morusin are crucial in molecular recognition of the target protein. Molecular modeling of proteins and docking analysis was utilized to screen for ligands of GABA transporter 1. In this study, morusin was identified as the strongest potential ligand for GABA transporter 1, in which Tyr140 and Ser396 of GABA transporter 1 may be involved in hydrogen bonding with morusin [23].

We briefly mentioned the chemical properties of morusin here, since this review mainly focuses on the biological aspects of morusin treatment with various clinical relevance. More detailed physiochemical properties of flavonoids including morusin and their purification processes are well described elsewhere [3,6,7].

### 2.2. The Effects of Morusin Treatment on Cellular Processes

**Figure 2:**
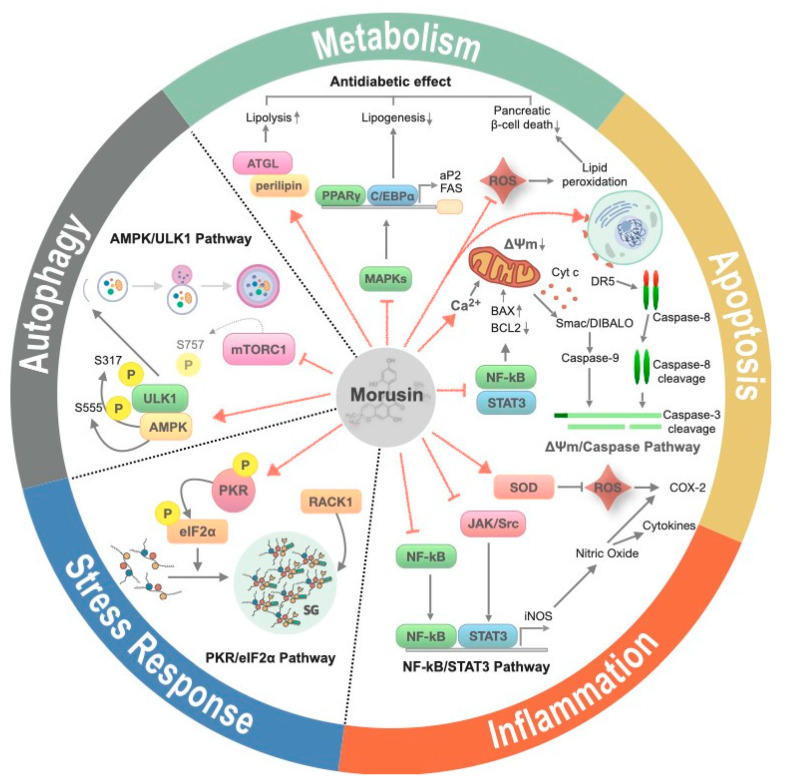
The effects of morusin on cellular processes. Morusin elicits various cellular processes including suppression of inflammation, induction of apoptosis, autophagy and stress granule formation and is involved in the homeostasis of glucose and lipid metabolism. Red solid arrow and solid bar indicates activation and suppression of target molecule, respectively. Upwards and downwards arrow indicates an increase and decrease of target protein or pathway, respectively.

Multiple physiological processes have been proposed as effectors of morusin treatment in various biochemical studies (Figure 2). In this section, we describe the cellular processes specifically implicated in morusin-targeted human pathophysiology discussed later on.

#### 2.2.1. Inflammation

Many studies have confirmed the anti-inflammatory capacity of morusin in different in vivo and in vitro contexts—(1) Morusin treatment inhibits secretion of cytokines such as CCL5 and CCL17 in TNFα- and IFN-γ-stimulated keratinocytes and also inhibits the release of histamine and LTC_4_ in A23187-stimulated MC/9 mast cells [24]. (2) Morusin treatment inhibits PMA-induced MUC5AC production in a human pulmonary mucoepidermoid cell line NCI-H292, showing prominent anti-inflammatory effects in vitro [25]. (3) Morusin treatment ameliorates IL-1β-induced chondrocyte inflammation and abrogates osteoarthritis in destabilization of the medial meniscus model in vivo [26]. (4) Morusin treatment inhibits NO production from LPS-induced RAW264.7 cells [27]. (5) Morusin treatment protects against 2,4,6-trinitrobenzensulfonic acid (TNBS)-induced colitis in rats [28]. (6) Morusin treatment alleviates inflammatory signaling thereby controlling the outgrowth of *mycoplasma pneumonia* [29].

This explosion of observations on the anti-inflammatory effects of morusin have raised questions of the molecular and biochemical targets of morusin and the underlying mechanisms. In this context, the antioxidant capacity of morusin has been linked to the suppression of iNOS induction, thereby reducing nitric oxide formation [27,30,31]. This mechanism may also be associated with the neuroprotective function of morusin against NO-induced cell death in SH-SY5Y cells [31]. Although the mechanism underlying iNOS regulation by morusin has not been directly addressed yet, it may be recapitulated by the NF-κB pathway, a key intracellular pathway that governs pro-inflammatory signaling in multiple ways including reactive oxygen species (ROS) production, cytokine production and immune cell activation [32,33]. Furthermore, morusin suppressed STAT1-mediated cytokine secretion in TNF-α- and IFN-γ-stimulated keratinocytes [24]. Indeed, many studies have already emphasized that the versatile effects of morusin may be associated with downregulation of the NF-κB pathway and its crosstalk with STAT1, STAT3 and Wnt/β-catenin signaling [24,25,26,29,34,35,36,37,38,39,40]. Given that activation of the NF-κB and STAT signaling pathways is one of the downstream events of EGF receptor signaling and since morusin directly binds to the catalytic domain of the EGF receptor for inactivation of EGFR [14], it is plausible that morusin-mediated blocking of EGFR signaling may contribute to the down-regulation of NF-κB-mediated iNOS induction and NO synthesis, as well as STAT1-mediated cytokine secretion, to result in anti-inflammatory effects. In addition, several in vitro studies argued that the anti-inflammatory effects of morusin are partly attributed to its role as an allosteric inhibitor of cyclooxygenase-2 (COX-2) and lipoxygenases (LOXs), which are key rate enzymes implicated in arachidonic acid metabolism [41]. Given that arachidonic acid is a key molecule spiking intracellular pro-inflammatory signaling followed by immune cell activation [42], such regulations may be clinically relevant to diseases associated with aberrant inflammation.

#### 2.2.2. Apoptosis

Although cytotoxicity upon morusin treatment has been observed across multiple cancer cell lines, biochemical characterization of the underlying mechanisms and clinical potential in vivo are still active areas of research. In particular, multiple recent studies have performed a thorough assessment of the biochemical targets of morusin, allowing for the identification of apoptosis as a critical tumor-killing effect of morusin.

Apoptosis is a type of tightly regulated programmed cell death, characterized by cell shrinkage, nuclear fragmentation, chromatin condensation and chromosomal DNA fragmentation [43]. Apoptosis can be subclassified into extrinsic and intrinsic pathways according to the origin of stimuli (intracellular vs extracellular signals). This biological process has been appreciated as an attractive druggable target in numerous pathological contexts especially including cancer [44]. Morusin effects on apoptosis in cancer cells has been mostly linked to its capacity to suppress the NF-κB pathway, since suppressors of the intrinsic apoptotic pathway, such as cIAP and Bcl-xL, are representative downstream targets of p50/p65, the core transcription factor of the NF-κB pathway [43]. The potential involvement of the NF-κB pathway was first suggested by a study in which morusin treatment led to inhibition of the NF-κB pathway and activation of intrinsic apoptosis in human colorectal cancer cells [35]. Such observations were also substantiated by studies across multiple cancer cell lines in which morusin treatment resulted in concomitant NF-κB pathway inhibition and activation of apoptosis [40,45,46].

In addition, several studies have suggested the possibility of STAT3 as a component of the mechanisms underlying the pro-apoptotic effects of morusin [14,36,38,47,48], which may be relevant since (1) ROS formation is both a consequence and a driving force of STAT3 activation [49] and (2) the STAT3 and NF-κB pathways synergistically promote transcriptional activation of suppressors of the intrinsic apoptotic pathway [50]. Consistently, STAT3 target genes, such as anti-apoptotic genes encoding Bcl-xL, Bcl-2, XIAP, survivin and cell cycle regulators (c-Myc and cyclin D), are down-regulated upon morusin treatment, while pro-apoptotic Bax expression was induced [36,38,40,47,51]. In addition, the administration of morusin reduces mitochondrial membrane potential resulting in the release of cytochrome c and Smac/DIABLO and thus, apoptosis is facilitated by the activation of caspase-9 and caspase-3 [35,48,52].

Turning off signaling pathways implicated in cell survival and proliferation has been functionally associated with the activation of apoptosis [53]. In this regard, morusin also exerts its pro-apoptotic effects by inhibiting relevant pathways including the PI3K-AKT and MAPK signaling pathways [35,54,55]. However, it is still unclear if such mechanisms can be dissociated from morusin effects on the NF-κB pathway, which has cross-talk with the PI3K-AKT, MAPK pathways in many physiological and pathological contexts [54,55].

Finally, some evidence has suggested that morusin treatment also results in the activation of extrinsic apoptotic pathways, in which morusin strongly increased expression of the death receptor DR5 at the transcriptional level and conferred sensitization of glioblastoma to TRAIL signaling [35,38]. However, thorough assessment of the underlying mechanisms may be further required to interpret morusin effects on the extrinsic apoptotic pathways, considering the intimate crosstalk between the intrinsic and extrinsic apoptotic pathways [56].

#### 2.2.3. Metabolism

Metabolism plays key roles in energy homeostasis and signal transduction, which are fundamental aspects of organisms. One of the unique aspects of metabolism is the remarkable metabolic flexibility in response to various stimuli [57]; metabolic pathways are dramatically rewired as an adaptive response to specific physiological contexts such as starvation and energy overload. Such adaptations have also been proposed as either the consequence or cause of numerous human pathologies including metabolic disorders, cardiovascular diseases and cancer.

Morusin treatment was shown to exert beneficial effects on systemic and cellular metabolism. Those effects have also been mostly associated with the antioxidant capacity of morusin. Multiple studies have shown that morusin treatment reduces reactive oxygen species in metabolically relevant organs, in which redox homeostasis is tightly associated with general metabolic fitness including increased mitochondrial performance and the amelioration of dysregulated fuel metabolism [58]. For example, treatment of *Morus alba* root bark extract including morusin reduces lipid peroxidation, thereby leading to hypoglycemic effects in vivo in a streptozotocin-induced mouse model of type I diabetes. Such beneficial effects may be associated with decreasing oxidative stress and preservation of pancreatic β-cell integrity by reducing cell death [59,60].

It has recently been shown that morusin also serves as an allosteric inhibitor of metabolic enzymes including pancreatic lipase, UDP-glucuronosyltransferase (UGT) and cytochrome p450 (CYP450) [16,61]. Such findings may be clinically relevant, since these enzymes have been implicated in the dysregulation of lipid homeostasis and endoplasmic reticulum stress, which play key roles in the pathogenesis of various metabolic disorders including diabetes [62,63]. Of note, CYP450 and other ER-resident lipid oxygenases including CYP1A2, CYP2C9, CYP2D6, CYP2E1, CYP3A4 and CYP2C19 have been proposed as metabolizing enzymes of morusin *per se* [17,20], although it is unclear whether the mechanisms contribute to the drug resistance or whether the intermediates mediate the beneficial effects of morusin.

Morusin has also been highlighted as a potent *de novo* lipogenesis inhibitor as well as a lipolysis stimulator [64]. The study was performed in 3T3-L1 and primary adipocytes in which morusin treatment leads to reduced lipid build-up and increased lipid breakdown. Such observations may be associated with changes in multiple processes including down-regulation of the MAPK pathway downstream of insulin receptor signaling, adipogenic transcription factors (PPARγand C/EBPα) and lipogenic factors (aP2 and FAS), while expression of lipolytic factors (HSL, ATGL and perilipin) are enhanced by morusin administration in differentiated 3T3-L1 adipocytes. However, the trans-differentiation of glioblastoma multiforme (GBM) cancer stem cells into adipocyte-like cells was induced by morusin treatment, in which the expression of adipogenic proteins including PPARγ, Adipsin D and aP2 were enhanced in a dose-dependent manner [39]. A similar result was observed with breast cancer cells in which differentiation of breast cancer cells into adipocyte-like cells was induced by morusin treatment and concomitantly expression of adipogenic protein was increased [65]. The detailed clinical potential of morusin treatment for metabolic disorders including diabetes has been discussed in a later section.

#### 2.2.4. Autophagy

**Figure 3:**
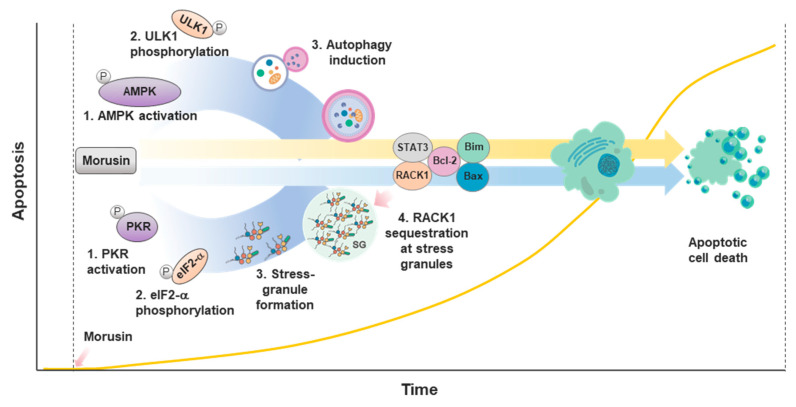
Morusin-induced autophagy and stress granule (SG) formation inhibits induction of apoptosis at early times of the stress response. Morusin induces apoptosis, autophagy and SG formation. However, induction of autophagy and SG formation occurs ahead of apoptosis induction at the early times of morusin treatment, resulting in a delay of the induction of apoptosis.

Autophagy is a physiological process through which unnecessary and dysfunctional cellular components are removed or recycled [66]. Various extracellular and intracellular signals induce autophagy and thus autophagy plays a key role in the intricate regulation of cellular homeostasis including cell fate determination, fuel metabolism and mitochondrial fitness. In this context, autophagy has been implicated in numerus human pathologies particularly associated with energy stress and mitochondrial dysfunction, such as cancer and neurodegeneration, respectively. This notion, together with previous reports in which drug resistance of cancer cells are tightly associated with the capacity of cells to induce autophagy [67], brought us to the question as to whether cancer cells employ autophagy as a protective mechanism against morusin-induced cell death. Biochemical evidence in our study showed that morusin treatment leads to mTOR1 inhibition and the subsequent activation of AMPK, resulting in ULK1-mediated autophagy activation. Such autophagy activation is associated with the reduced apoptosis induced by morusin (Figure 3), corroborating the idea that activation of autophagy confers drug resistance to cancer cells [68]. This mechanism may be noteworthy in the context of the therapeutic relevance of morusin for neurological disorders, given the anti-cell death effects of morusin on neuronal cells [31] and the intimate connection between autophagy and neurodegenerative diseases [69].

#### 2.2.5. Stress Response

Cells have evolved multiple physiological strategies to cope with diverse environmental and intracellular stresses. Among these is stress granule (SG) formation, during which macromolecular aggregates of mRNA and proteins are assembled, leading to the attenuation of translation and activation of various physiological pathways for DNA damage repair and cell survival [70]. Since study of the SGs has mainly focused on biochemical or mechanistic perspectives, the physiological and pathological relevance of the SGs is still enigmatic. Recent in vitro and in vivo clinical evidence has proposed the potential implication of SGs in cancers and neurodegenerative diseases, warranting a more detailed examination of the underlying mechanisms [71,72]. In particular, the SGs are induced by various types of cytotoxic drugs, thereby contributing to drug resistance [73]. Our recent study showed that SG formation is induced by morusin treatment [74]. PKR was identified as a target of morusin in an un-biased systemic phospho-antibody array. PKR activation and subsequent eIF2α phosphorylation are key biochemical events for morusin-induced SG formation, which leads to the retention of RACK1 within the SGs. Since RACK1 is a pro-apoptotic molecule that activates intrinsic apoptotic pathways and together with our data in which RACK1 manipulation decouples SG formation and morusin-induced cell death, we suggested that morusin-mediated cell death may at least in part be attributed to RACK1 activation (Figure 3).

Although our study only highlighted the potential connection between SG formation and the intrinsic apoptotic pathway, it may also be of interest to study the biochemical mechanism(s) and the pathological relevance of interplay with other pathways including the extrinsic apoptotic pathways also activated by morusin, as well as inflammatory signaling, given the involvement of PKR in this mechanism.

### 2.3. Clinical Potential of Morusin Treatment in Pathophysiology

Exploding number of causality studies linking specific cellular processes to human pathologies, together with morusin implication in the processes, warranted assessment of the clinical potential of morusin in various disease settings in vivo and in vitro. Here, we discuss recent advances in the understanding of the potential application of morusin in treating common human diseases.

#### 2.3.1. Inflammatory Diseases

The anti-inflammatory capacity of morusin has been tested in various models of inflammatory diseases, which was initiated with several in vitro studies on morusin-mediated inhibition of iNOS, cyclooxygenases and lipoxygenases [27,41]—(1) Lee et al. demonstrated that morusin reduces musin secretion in NCI-H292 cancer cells with mucoepidermoid characteristics following chronic inflammation [25]. This in vitro observation was concordant with in vivo results in which morusin treatment alleviates the hypersecretion of airway mucin in a rat model of bronchitis induced by sulfur dioxide treatment. (2) Morusin displays potential anti-allergic and anti-inflammatory effects on atopic dermatitis, a common chronic inflammatory skin disease, which was well-demonstrated by Jin et al., in their in vitro study with MC/9 mast cells and HaCat keratinocytes [24]. Mechanistically, they proposed the dual roles of morusin in modulating inflammatory signals in both keratinocytes and immune cells via STAT1/NF-κB and lipoxygenase, respectively. (3) Morusin treatment inhibits the NF-κB signaling pathway and thereby dampens IL-1β-induced chondrocyte inflammation and osteoarthritis using mouse chondrocytes and destabilization of the medial meniscus (DMM) model, a mouse model of osteoarthritis [26]. (4) Morusin treatment has been tested in a chemical-induced rat model of colitis. Histological observations following morusin gavage in the rats showed a significant reduction of tissue damage score and pro-inflammatory markers (TGF-β1 and IL-1β) and an increase in the level of antioxidant enzymes (superoxide dismutase and catalase), indicating that morusin may have therapeutic potential in treating inflammatory bowel disease [28].

Numerous microbes and viruses take advantage of host cellular processes and machineries to maximize their infection capacities. One such strategy is to provoke cellular and systemic inflammatory responses. Morusin has been administered to a few models of infectious diseases, such as an in vivo mouse model of mycoplasma pneumonia infection, in which morusin treatment efficiently suppressed mycoplasma pneumonia via the inhibition of Wnt/β-catenin and NF-κB signaling [29]. Morusin also appears to exert anti-microbial and anti-viral activities on microorganisms *per se*; Pang et al. and We et al., demonstrated that morusin has the remarkable capacity to target *Staphylococcus aureus* and clinical methicillin-resistant *Staphylococcus aureus* (MRSA) in vitro and in vivo [75,76]. The mechanism of action may be associated with the chemical properties of morusin as an isopentenyl leading to an increase in membrane permeability, inhibition of the phospholipid-repair system and dissipation of the proton motive force of the bacteria [77]. Mechanisms underlying the anti-viral effects of morusin are less clear. One possible explanation may be that morusin exerts its effects by allosteric inhibition of essential viral components. Of note, an in silico analysis proposed that morusin could directly inhibit HIV-1 reverse transcriptase activity [78] and SARS-CoV-2 protease [79].

#### 2.3.2. Neurological Disorders

In Chinese medicine, *Morus alba* has been used as a neuroprotective herb. The neuroprotective functions of morusin and the implications for disease have recently been appreciated at the cellular and molecular levels. For example, morusin treatment protects neuroblastoma SH-SY5Y cells from nitric oxide-induced cell death [31]. This in vitro observation was also recapitulated by a follow-up study in which the in vivo relevance of the antioxidant capacities of morusin in neuroprotection was tested [80]. In the study, authors used a rat model of memory disorder induced by aluminum trichloride (AlCl_3_), showing that morusin ameliorates the impaired memory and learning capacity by decreasing the AlCl_3_-induced rise in brain acetylcholinesterase (AChE) activity and brain oxidative stress levels. Although in vivo studies of the effects of morusin on neurological disorders are very limited, morusin may exert beneficial functions against neurodegenerative diseases including Alzheimer’s disease, given that (1) morusin shows inhibitory activity against AChE, butyrylcholinesterase (BChE) and β-site amyloid precursor protein cleaving enzyme 1 (BACE1), which play important roles in the prevention and treatment of Alzheimer’s disease [18,81] and (2) morusin treatment activates autophagy in normal cells and the dysregulation of autophagy significantly contributes to the pathogenesis of neurodegenerative disorders [68,69]. Accordingly, an integrative approach of pharmacokinetics and a structural bioinformatics approach of 210 plant compounds revealed that morusin displays high potential as an anti-Alzheimer drug [82], corroborating the idea of the clinical potential of morusin in targeting neurodegenerative diseases.

#### 2.3.3. Diabetes

Diabetes mellitus is a pathological condition in which the body displays impaired capacity to maintain coordinated systemic fuel metabolism upon various nutrient perturbations, particularly perturbations of glucose, due to (1) pancreatic β-cell dysfunction followed by reduced serum levels of insulin (Type I diabetes), and/or (2) reduced insulin sensitivity of systemic cells and relatively reduced insulin secretion from pancreatic β-cells (Type II diabetes) [83]. Although numerous factors have been implicated in such confounding metabolic disorders, the causalities and underlying mechanisms are not fully understood. Among these, the reduced lipid storage capacity of adipocytes and aberrant lipid metabolism with ROS accumulation in metabolically relevant organs including adipocytes, liver, muscle and pancreatic β-cells have been tightly associated with the pathogenesis of the disease [84,85]. In this context, multiple in vivo and in vitro evidence linked the versatile effects of morusin on the metabolic pathways to its clinical potential in treating diabetes. For example, two independent studies showed that morusin treatment ameliorates hyperglycemia and dysregulated lipid homeostasis in a mice model of type I diabetes induced by streptozotocin treatment that specifically kills pancreatic β-cells [59,60]. Such mechanisms may involve morusin-mediated alleviation of the peroxidation of lipid species spilled over from adipocytes, due to insulin secretion levels insufficient to maintain the fat storage capacity of adipocytes.

Although no studies have yet tested the effects of morusin on the pathogenesis of type II diabetes using relevant in vivo models (e.g., *ob/ob*, *db/db* mouse or HFD-fed mice)*,* morusin may also exert anti-diabetes effects in this context, considering that morusin potentially regulates systemic ROS levels, lipogenesis, lipolysis, C/EBPβ and PPARγ signaling, arachidonic acid metabolism and ER-resident proteins implicated in ER stress [16,59,64], all of which have been frequently implicated in type II diabetes as well as type I diabetes [42,63,86,87].

#### 2.3.4. Cancer

The implication of morusin in multiple biological pathways that cancers take advantage of, described above, led to many studies testing the antitumoral capacities of morusin in vitro, with recent initiation of research on its clinical potential in vivo. Indeed, very early studies on the chemical properties of morusin already appreciated its antitumoral potential [6], connecting the strong antioxidant capacity of morusin to the aberrant ROS production broadly relevant to the pathogenesis of cancers [88].

The effects of morusin on specific subtypes of cancers and the underlying biochemical mechanisms have recently been studied using various in vitro and in vivo model systems (Table 1). For example, Wang et al., demonstrated the clinical potential of morusin in targeting the stemness capacity of human cervical carcinoma in vitro [40]. Such an approach may be relevant, as NF-κB is a well-established upstream transcription factor of genes for stemness and metastatic capacities of various cancers including cervical cancers [33]. In this study, morusin treatment was shown to decrease the expression of Oct4, SOX2, ALDH1, as well as epithelial-to-mesenchymal markers, thereby decreasing the stemness signature of the cancer cells. Morusin-mediated inhibition of the NF-κB pathway also leads to reduced expression of Bcl-2 and upregulation of pro-apoptotic proteins including Bax and caspase-3, which results in the apoptotic death of the cancer cells. Morusin may also display antitumor effects independent of its capacity to activate apoptosis at non-cytotoxic concentrations (lower than 10 μM), since morusin promotes VDAC-mediated Ca^2+^ influx into mitochondria leading to mitochondrial Ca^2+^ overload and mitochondrial dysfunction and subsequent paraptosis-like cell death observed in an in vitro and in vivo model of epithelial ovarian cancer with apoptotic resistance [37].

Morusin treatment exerts beneficial effects against liver cancers as well. The intravenous injection of morusin in a xenograft mouse model of hepatocarcinoma significantly reduced tumor growth without any side effects. The histological and biochemical evidence provided in this study indicated that the mechanism may be associated with the activation of p53, survivin, cyclin B1 and caspase-3 and a decrease in the expression of NF-κB [47]. Moreover, morusin treatment also suppresses the metastatic capacity of the human hepatoma SK-Hep1 cell line, which may be biochemically associated with inhibition of STAT3 and NF-κB followed by the reduction of the expression of metastatic markers, such as vimentin, α2- and β1-integrins [34]. Such in vitro observations were also confirmed in an experiment with immunocompromised mice xenografted with SK-Hep1 cells, in which morusin treatment significantly decreased lung colonization of the cancer cells. Following the report that morusin effectively inhibits the growth of tumors in mice transplanted with H22 liver tumor cells [46], similar inhibitory effects of morusin on tumor xenografts were observed for liver, ovary, breast, kidney and gastric cancer cells [37,47,54,65,90]. In addition to inhibitory effects on tumor growth, tube formation of human umbilical vein endothelial cells (HUVEC) and expression of angiogenesis-related genes were also inhibited by morusin treatment in vitro [47,52], suggesting the anti-metastatic potential of morusin.

Morusin also targets the STAT3 pathway and its downstream targets, including survivin, cyclin B1, to exert antitumor activity in prostate and pancreatic cancers [36,48]. In the case of pancreatic cancer, pancreatic lipase may be another relevant target of morusin, considering (1) the strong association between pancreatic cancer and the levels of pancreatic lipase [48] and (2) the potential role of morusin in the allosteric regulation of pancreatic lipase [61].

The antitumor capacity of morusin has also been tested in glioblastoma. For example, the stemness capacity of glioblastoma multiforme (GBM) cancer stem cells (GSCs) were targeted by morusin treatment in vivo and in vitro [39]. This observation may be mechanistically attributable to the expression of genes downstream of NF-κB, which was already proposed in the previously described study with the cervical carcinoma model [40]. Of note, the reduced stemness of the cancers upon morusin treatment results in adipocyte-like trans-differentiation, followed by activation of intrinsic apoptosis [39]. The underlying mechanisms may also involve the activation of extrinsic apoptosis, as morusin treatment promotes expression of an extrinsic receptor, Death receptor 5, thereby sensitizing the glioblastoma to TRAIL, an antitumor chemical that mimics ligands for the extrinsic receptor [38].

Morusin may also employ other various cellular processes independent of NF-κB for targeting cancers—(1) An in vitro study of gastric cancer showed that morusin governs the expression of c-Myc and multiple downstream genes including CDKs and cyclins implicated in cell proliferation, thereby exerting antitumor capacity [90]. (2) The PI3K/AKT pathway has been proposed as a target of morusin in treating osteosarcoma in vitro [55]. (3) Morusin may directly target the MAPK pathway in renal carcinoma in vitro, although this should be further validated considering the intimate crosstalk between the NF-κB and MAPK pathway [54]. (4) C/EBPβ- and PPARγ-mediated lipoapoptosis may be therapeutically relevant targets of morusin in breast cancers, proposed in a study using multiple breast cancer cell lines and a xenograft model of breast cancers [64]. This may be of great importance considering that the capacity of mitochondrial fatty acid oxidation and fatty acid availability associated with host metabolism have recently emerged as key factors contributing to the pathogenesis of breast cancers [91,92]. (5) Studies with a panel of lung cancer cell lines showed that morusin treatment not only modulates conventional targets including NF-κB, STAT3 and downstream genes such as VEGF but may also directly bind and dephosphorylate EGFR on an active site, which would contribute to the further reduction of STAT3/NF-κB activity [14].

A cancer cell continuously undergoes dynamic remodeling of numerous cellular processes, which confer various benefits such as drug resistance. In this context, the two recent studies from our lab have highlighted the biochemical mechanisms by which cancer cells gain resistance against the cytotoxicity of morusin [68,74]. As described in the previous section, cancer cells promote autophagy and SG formation in response to morusin treatment, thereby desensitizing the apoptotic signals exerted by morusin treatment. Indeed, autophagy and SGs have already been proposed as the ‘shield pathways’ of cancers against various drug treatments. This notion, together with multiple biochemical studies in which morusin synergized with inhibitors of either autophagy or SG formation to boost its antitumor capacity, proposed the cotreatment of morusin and the inhibitors as a therapeutically relevant strategy for targeting cancers with drug resistance. However, whether such resistant mechanisms are still relevant in the context of various subtypes of cancers in vivo should be addressed.

## 3. Conclusions

The well-acknowledged functions of the root bark of *Morus* in Chinese herbal medicine have been expanded to the potential application of individual phytochemicals extracted from such arcane plants to treat multiple chronic diseases common in modern society. Morusin has been appreciated as a phytochemical with the unique physiochemical properties of isoprene flavonoids, allowing for versatile salutary effects including antioxidant and tumor killing activities. Morusin reduces ROS formation and inflammation at non-cytotoxic concentrations (lower than 10 μM), while it induces ROS formation and cytotoxicity against cancer cells at high concentrations (higher than 20 μM). A growing body of evidence, described here, highlighted the biological properties of morusin in multiple in vitro and in vivo pathophysiological settings and proposed clinical potential. The as yet unraveled clinical potential of morusin is waiting for thorough scientific assessment of the effects of morusin on human pathology with an accompanying understanding of the underlying biochemical mechanisms and effective platforms, which may allow for *bona fide* application of morusin in patients with various pathologies in the near future.

## Figures and Tables

**Table 1:** The Effects of Morusin on Subtypes of Cancer.

Organ	Cell Type	Effects on Cellular Processes	System	Reference
Liver	LO2 HepG2 Hep3B	Apoptosis induction & angiogenesis inhibition increases the expression of caspase-3 and the Bax/Bcl-2 expression ratio inhibits tube formation of HUVECs in vitro and suppresses constitutive as well as IL-6-induced STAT3 phosphorylation	in vitro in vivo	[47]
Liver	SK-Hep1	Anti-tumor progression through suppressing STAT3 and NF-kB suppresses cell-matrix adhesion, cell motility and cell invasion at non-cytotoxic concentration increases the expression of E-cadherin and decreases the expression of vimentin and α2-, α6-, β1-integrin	in vitro in vivo	[34]
Lung	A549	Apoptosis induction & cell migration suppression induces apoptosis by loss of mitochondrial function and increases the antioxidant activities by up-regulation of SOD inhibits the invasion and migration by down-regulation of COX2 and VEGF at the transcription level	in vitro	[52]
Lung	H1299 H460 H292	Apoptosis induction by suppression of EGFR/STAT3 activation inhibits phosphorylation of EGFR and STAT3 and shows potential to treat advanced NSCLC with acquired resistance to EGFR tyrosine kinase inhibitor The docking analysis: morusin directly binds to the tyrosine kinase domain of EGFR	in vitro	[14]
Breast	MCF-10A 4T1 MCF-7	Suppression of cancer cell growth through C/EBPβ- and PPARγ-mediated lipoapoptosis inhibits human breast cancer cell proliferation and increases the expression of C/EBPβ, PPARγ, adipsin D and perilipin induces adipogenic differentiation, apoptosis and lipoapoptosis of cancer cells	in vitro in vivo	[65]
Breast	MCF-10A MCF-7 MDA-MB231	Apoptosis induction The apoptosis marker proteins, cleaved caspase-3 and caspase-9 were consistently upregulated suppresses the expression of the anti-apoptotic Survivin and induces pro-apoptotic Bax expression	in vitro	[51]
Brain	U87 GI-1 HCN-1A	Morusin-loaded nanoparticles for targeted glioblastoma therapy Morusin was loaded in chlorotoxin-modified PLGA nanoparticles which target chloride channels and MMP-2 in glioma tumor cells Morusin-loaded nanoparticles inhibit growth of U87 and GI-1 glioma cells by ROS generation, enhanced caspase activity, cytoskeletal destabilization and inhibition of MMP activity	in vitro	[89]
Brain	WJ1 WJ2	Inhibition of glioblastoma stem cell growth through stemness attenuation, adipocyte transdifferentiation increases adipogenic markers, such as PPARγ, adipsin D, aP2 and perilipin and induces apoptosis reduces stemness of GSCs by inhibition of the expression of stemness markers (CD133, nestin, Sox2 and Oct4)	in vitro in vivo	[39]
Brain	U251MG LN18 U87MG	TRAIL sensitization by regulating EGFR and DR5 in human glioblastoma cells Combinatorial treatment of TRAIL with morusin synergistically decreased cell viability and increased apoptosis induces the expression of DR5 and decreases anti-apoptotic survivin and XIAP by reduced expression of EGFR and pSTAT3	in vitro	[38]
Skin	JB6 P+	Blocking TPA-induced malignant transformation of mouse epidermal cells reduces the TPA-induced ROS production, AP1 and NF-κB in JB6 P+ cells at non-cytotoxic concentration decreases TPA-upregulation of COX-2, N-cadherin and Vimentin	in vitro	[45]
Stomach	MKN45 SGC7901	Inhibition of cell proliferation and tumor growth by down-regulating c-Myc suppresses tumor growth and down-regulates CDKs and cyclins, such as CDK2, CDK4, cyclin D1 and cyclin E1. reduces the expression of c-Myc and c-Myc protein binding at the E-Box regions	in vitro in vivo	[90]
Pancreas	AsPC-1 BxPC-3 MIAPaCa-2 PANC-1	Apoptosis induction and inhibition of invasion by blockage of STAT3 signaling pathway inhibits STAT3 activation and suppresses activation of upstream JAK1, JAK2 and c-Src kinases. arrest cell cycle at G1/G0 or G2/M phase and causes induction of apoptosis and loss of mitochondrial membrane potential	in vitro	[48]
Bone	U2OS HOS	Inhibition of human osteosarcoma via PI3K-AKT signaling pathway promotes apoptosis and reduces the migration and invasion of osteosarcoma inhibits the PI3K/AKT signaling pathway and induces the expression of caspase-3 and caspase-8	in vitro	[55]
Ovary	A2780 SKOV-3 HO-8910	Paraptosis-like cell death induction through mitochondrial calcium overload and dysfunction causes mitochondrial Ca^2+^ influx and induces paraptosis-like cell death via mitochondrial Ca^2+^ overload increases ROS and decreases mitochondrial membrane potential and inhibits the growth of SKOV-3 xenograft in nude mice	in vitro in vivo	[37]
Prostate	DU145 PC-3 LNCaP RWPE-1	Cell death induction through inactivating STAT3 signaling reduces STAT3 activity by suppressing kinase activities of JAK2 and Src and increases SHP1 phosphatase activity down-regulates the expression of STAT3 target genes encoding Bcl-xL, Bcl-2, Survivin, c-Myc and cyclin D1	in vitro	[36]
Cervix	HeLa	Apoptosis induction & inhibition of human cervical cancer stem cell growth and migration through attenuation of NF-κB activity decreases the proliferation, tumor sphere formation and migration of human cervical CSCs and increases apoptosis decreases the expression levels of NF-κB/p65 and Bcl-2, while increases expression levels of Bax and caspase-3	in vitro	[40]
Kidney	769-P 786-O OSRC-2	Anti-cancer activity by disturbing MAPK signaling pathways inhibits cell growth and migration, induces cell apoptosis and induces the cell cycle arrest in the G1 phase up-regulates P-p38 and P-JNK levels, while the down-regulates P-ERK level	in vitro in vivo	[54]
Liver Spleen	H22	Inhibition of transplanted H22 hepatocarcinoma inhibits the tumor growth of transplanted H22 hepatocarcinoma in mice by reducing the expression of NF-κB increases the expression of p53, Survivin, cyclin B1 and caspase-3	in vitro in vivo	[46]
Colon Liver Breast	HT-29 Hep3B MCF-7	Apoptosis induction & suppression of NF-κB activity inhibits the phosphorylation of IKK-α, IKK-β and IκB-α and suppresses NF-κB nuclear localization and its DNA binding causes activation of caspase-8, change of mitochondrial membrane potential, release of Cytochrome c and Smac/DIABLO and activation of caspase-9 and caspase-3	in vitro	[35]
Cervix Breast Bone	HeLa U2OS ZR75B	Attenuation of RACK1-mediated apoptotic cell death by stress granule (SG) formation induces activation of PKR and subsequent eIF2α phosphorylation for SG formation sequestration of RACK1 within the SGs contributes to protection of cells from cell death	in vitro	[74]
Cervix Breast Bone Colon	HeLa MCF-7 U2OS HCT116	Autophagy induction inhibits cell death induces AMPK activation and inhibits mTOR activity, resulting in LC3-II accumulation and ULK1 activation for autophagy autophagy induction is an impediment for morusin-induced apoptosis	in vitro	[68]
